# Adjustable Vocal Implant System (VOIS) for Unilateral Vocal Fold Paralysis: Short‐Term Outcomes From a 12‐Patient Case Series

**DOI:** 10.1111/coa.70142

**Published:** 2026-07-16

**Authors:** Sam Kebbe, Michael Keane, Chuanyu Gao, Yadsan Devabalan, Guan‐Min Ho, Rohan Bidaye

**Affiliations:** ^1^ Leicester Medical School, George Davies Centre Leicester UK; ^2^ Northampton General Hospital Northampton UK; ^3^ Mackay Memorial Hospital Taipei Taiwan; ^4^ Yomin ENT & Pediatric Clinic New Taipei City Taiwan; ^5^ APrevent Medical Taipei Taiwan

**Keywords:** adjustable VOIS implant, medialisation thyroplasty, patient reported outcome measure, phonation outcomes, unilateral vocal cord palsy

## Abstract

**Background:**

Unilateral vocal cord palsy (UVCP) significantly affects phonation and swallowing. While injection laryngoplasty and medialisation thyroplasty are effective, both may require revision. Vocal Implant System (VOIS; APrevent Biotech GmbH) is an adjustable titanium‐silicone thyroplasty implant that allows postoperative modification, potentially reducing the need for further surgery.

**Methods:**

Twelve patients, seven male and five female, underwent VOIS implantation. Voice outcomes were assessed pre‐ and postoperatively using the GRBAS scale and the Voice Handicap Index‐10 (VHI‐10).

**Results:**

The composite GRBAS score improved significantly from 9.0 ± 1.87 to 3.75 ± 2.49 (*p* = 0.0024). The VHI‐10 decreased from 25.75 ± 9.36 to 14.92 ± 9.55 (*p* = 0.0093). No intra‐ or postoperative complications occurred. Two patients required postoperative implant volume adjustments—one performed in clinic and one under local anaesthetic in theatre.

**Conclusion:**

The VOIS implant thyroplasty demonstrated significant improvement in both perceptual and patient‐reported voice outcomes with a favourable safety profile. Larger, long‐term studies are warranted to confirm its comparative efficacy and durability.

## Summary

- The Vocal Implant System (VOIS) is a novel laryngeal implant that can be adjusted intra‐ and postoperatively to optimise the management of unilateral vocal cord palsies and their effects on phonation, swallowing, and quality of life.
- In this UK‐based case series, 12 patients received the VOIS implant, and their voice was assessed using the GRBAS and VHI‐10 scales.
- Objective assessment by clinicians found a statistically significant improvement in the GRBAS score.
- There was also statistically significant improvement in the patient‐reported VHI‐10 scores, exceeding the minimal clinically important difference.
- There were no intra‐ or postoperative complications, and only two patients required adjustment of the implant volume.

## 1 Introduction

Unilateral vocal cord palsy (UVCP) has a significant impact on quality of life primarily through its effect on verbal communication, swallowing and aspiration, and significant long‐term psychological distress [1]. Patients experiencing recurrent laryngeal nerve (RLN) injury will often present with breathlessness, hoarseness, impaired glottic closure leading to altered phonation and an increased risk of aspiration [1]. Recent estimates of its incidence in the UK are 5.13 per 1000 people per year [2]. Recurrent laryngeal nerve (RLN) injury is the primary cause of vocal cord palsy, most commonly due to iatrogenic damage during thyroid, parathyroid, or carotid surgery [3]. Other pathologies include head and neck neoplasms, neurological disorders, and infection [3].

A number of different procedures have been shown to be beneficial for UVCP. The most commonly used treatments include injection laryngoplasty (IL), medialisation thyroplasty, arytenoid adduction and laryngeal reinnervation. While studies have shown benefits to all of these, there is limited high‐quality evidence of superiority between any technique [4].

Injection laryngoplasty involves injection of a filler material into the paraglottic space, medializing the paralysed vocal cord. Various materials have been used including autologous fat, hyaluronic acid, collagen derivatives and calcium hydroxylapatite. IL has been used to treat UVCP and has been shown to effectively improve vocal function, either alone or in combination with vocal framework surgery [4]. However, there is reported variability in the duration of the improvements in vocal function. Some patients experience long‐term or even permanent benefits whilst others require multiple injections over the course of a lifetime due to reabsorption of the filler material [5]. Therefore, it is often used as a short‐term treatment for temporary causes of UVFP where there is the possibility of spontaneous recovery of vocal cord function [6].

For a more permanent solution, medialisation thyroplasty is commonly performed [7]. A number of implants of varying designs and materials have been designed, including silicone, Gore‐Tex and titanium. Studies have confirmed the long‐term improvement of voice quality and reduced rates of aspiration and subsequent pneumonia following medialization [4]. However, up to one‐third of patients may require revision for suboptimal correction [8]. Need for revision may be due to over‐ or under‐correction owing to the size of the implant. Other causes for revision include pre‐existing cord augmentation via injection laryngoplasty, mismatched vertical height of the vocal cords, persistent arytenoid adduction, implant migration, and laryngeal muscle atrophy secondary to paralysis leading to a deterioration in voice quality [8, 9, 10, 11].

The Vocal Implant System (VOIS; APrevent Biotech GmbH, Austria) represents an innovative thyroplasty implant to address UVCP. Preclinical data published by the creators of the implant outline the product roadmap beginning with in vivo trials on porcine models of UVFP, referring to human CT and MRI scans for approximating required sizes, and ex vivo acoustic and aerodynamic analyses. The implant is manufactured using titanium and silicone, two biologically inert materials routinely used in implantable medical devices. It is available in four sizes (extra small to large) differing in their length, width, and direction of expansion of silicone cushions [12]. Previous case series have demonstrated a significant improvement to both objective and subjective measures of vocal performance up to 1 year after implantation [13, 14].

This implant can be adjusted intra‐ and postoperatively by injecting saline directly into it. The variability of filling volumes caters to natural variation in laryngeal dimensions thus satisfying patient needs and optimising results. Furthermore, this customisation shortens the operation time, circumvents the constraints of laryngeal and surrounding soft tissue oedema during and after implantation, and reduces subsequent risks of airway compromise [13].

The VOIS implant was recently introduced at Northampton General Hospital, a large NHS district general hospital, for the treatment of UVCP. The objectives of this case series were twofold: (1) to assess changes in voice quality and patient satisfaction following VOIS implantation, and (2) to evaluate the clinical effectiveness of the device in a local UK patient cohort.

## 2 Materials and Methods

### 2.1 Patient Recruitment

Twelve patients underwent VOIS implantation at Northampton General Hospital between June 2024 and March 2025, with follow‐up until August 2025. All procedures were performed by the same consultant surgeon trained in thyroplasty surgeries.

### 2.2 Surgical Approach and Device Mechanism

Intraoperative sedation was used in ten patients with propofol and remifentanil delivered via target‐controlled infusion. Oxygen at 2–6 L/min was administered through nasal prongs with continuous capnography. A laryngeal mask airway was used in one patient, and a size‐5 microlaryngeal tube in another who underwent general anaesthesia.

Anatomical landmarks were used to determine the optimal window location. The midpoint between the thyroid notch and the inferior border of the thyroid cartilage (M1) and the midpoint of the posterior thyroid lamina (M2) were identified to establish a reference line. A cartilage ruler from the VOIS instrument set was then used along this reference line to determine the appropriate implant size (XS–L).

Additional landmarks, including the inferior thyroid tubercle (points A and B), were used to guide accurate marking of the superior and anterior borders of the thyroplasty window using a size‐specific stencil. After confirming the template position, the outer cortex of the thyroid cartilage was marked using monopolar cautery and drilled using the dedicated VOIS instrument set to create a thyroplasty window measuring approximately 11 × 6 mm. The inner perichondrium was preserved to minimise the risk of vocal fold injury. Particular care was taken during drilling along the inferior border of the window, where cartilage fractures may occur due to thinning of the thyroid cartilage.

The VOIS implant was then inserted with the expandable silicone cushion positioned within the paraglottic space and secured using the integrated titanium fixation plate. The design of the VOIS implant allows preferential medialisation of the posterior aspect of the vocal fold, as the expandable balloon is oriented posteriorly, enabling effective closure of the posterior glottic gap without requiring deliberate positioning adjustments during insertion.

Intraoperative flexible nasoendoscopy was performed to assess glottic closure, arytenoid position, and voice quality during phonation. Sedation levels were reduced where required to facilitate real‐time voice assessment. The VOIS implant contains an adjustable silicone balloon that can be expanded with saline to fine‐tune medialisation. Maximum recommended filling volumes are 0.17, 0.25, 0.30 and 0.30 mL for implant sizes XS, S, M and L respectively. Figure 1 includes a rendered image of the implant, as well as intraoperative photographs. A key advantage of the VOIS system is the ability to perform postoperative adjustments through the integrated injection port without requiring revision surgery.

**FIGURE 1:**
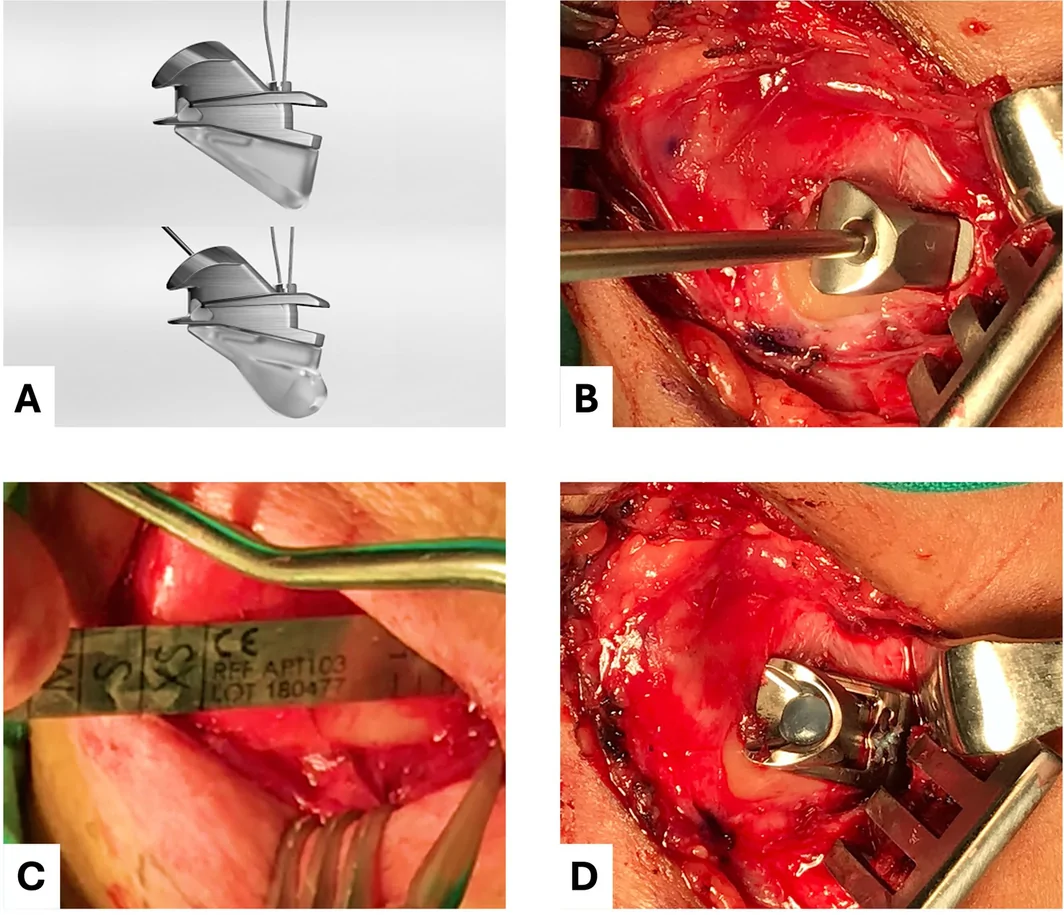
Rendered image of the VOIS implant and intraoperative photographs. (A) 3D animation of implant. (B) Measuring of thyroplasty window using a guide. (C) Size‐specific stencil to gauge the accurate size implant to use. (D) Implant in situ after final insertion.

The approximate cost of the VOIS implant used is around £3000 per device, although this may vary depending on institutional procurement agreements.

### 2.3 Ethics

This study involving human participants was conducted in accordance with the ethical standards of the institutional and national research committees and with the 1964 Declaration of Helsinki and its later amendments or comparable ethical standards. The study was approved by the ENT clinical governance team and registered with the departmental audit database (ENT/CA/2025‐15/08). Upon consenting for and booking the procedure, patients were informed that their anonymised clinical data, including pre‐ and postoperative assessments, may be used for research purposes to inform best practice. None of the patients included in this study objected to participation in research.

### 2.4 Data Collection and Interpretation

Two validated measures of vocal performance were collected. The first, the GRBAS scale, is a subjective score that assesses 5 different domains of a patient's voice: grade, roughness, breathiness, asthenia and strain [15]. It was assessed by a qualified speech and language therapist during pre and postoperative appointments between 1 to 5 months after the intervention. The second, the Voice Handicap Index‐10 (VHI‐10), is a validated tool developed from the previously used Voice Handicap Index score to assess the psychosocial impact of vocal conditions [16]. This was reported by patients after surgery. Patient records were reviewed retrospectively to gather demographic and clinical details, including age, sex, comorbidities, aetiology of palsy, and previous interventions.

Results are presented as mean values ± 1 standard deviation unless stated otherwise. *p*‐values below 0.05 were considered significant. Statistical analyses were performed in Microsoft Excel (Version 2508), GraphPad Prism (Version 10.4.2) and R (Version 4.5.1) accessed through RStudio (Posit Software, Version 2015.05.1). Figures were generated using the R package ggplot2 (Version 3.5.2). Data distribution was assessed using the Shapiro–Wilk test, which determined the majority of the data were not normally distributed. Due to the sample size (*n* = 12) and lack of normality, the non‐parametric Wilcoxon signed‐rank test was used to assess for a significant change in vocal performance postoperatively.

## 3 Results

### 3.1 Patient and Surgical Data

Twelve patients (seven male, five female) underwent VOIS implantation between June 2024 and March 2025 at Northampton General Hospital. The median age was 71.5 years (range: 42–81). The aetiologies of UVCP included iatrogenic injury (*n* = 8), intracranial infection (*n* = 1), lung malignancy (*n* = 1), stroke (*n* = 1), and idiopathic causes (*n* = 1). Nine patients (75%) had previously undergone IL, with five having received more than one procedure.

Of the seven male patients, six received M_3.5 implants and one received L_3.5 implants. All five female patients received XS implants.

### 3.2 Functional Voice Assessment

Objective vocal performance was assessed using the GRBAS score. All individual GRBAS domains showed significant postoperative improvement (Table 1). The composite GRBAS score decreased from 9.0 ± 1.87 to 3.75 ± 2.49 (Wilcoxon signed‐rank test, *p* = 0.0005) (Figure 2).

**TABLE 1:** Pre‐ and postoperative GRBAS scores for all patients (*n* = 12) reported as a mean of patient scores ±1 standard deviation; Wilcoxon signed rank test for comparison.

	Preoperative	Postoperative	*p*
Grade	2.08 (0.28)	1.08 (0.64)	0.0039
Roughness	2 (0.41)	1 (0.71)	0.0078
Breathiness	1.58 (0.76)	0.58 (0.49)	0.0156
Asthenia	1.67 (0.62)	0.5 (0.5)	0.0078
Strain	1.67 (0.47)	0.58 (0.64)	0.002

**FIGURE 2:**
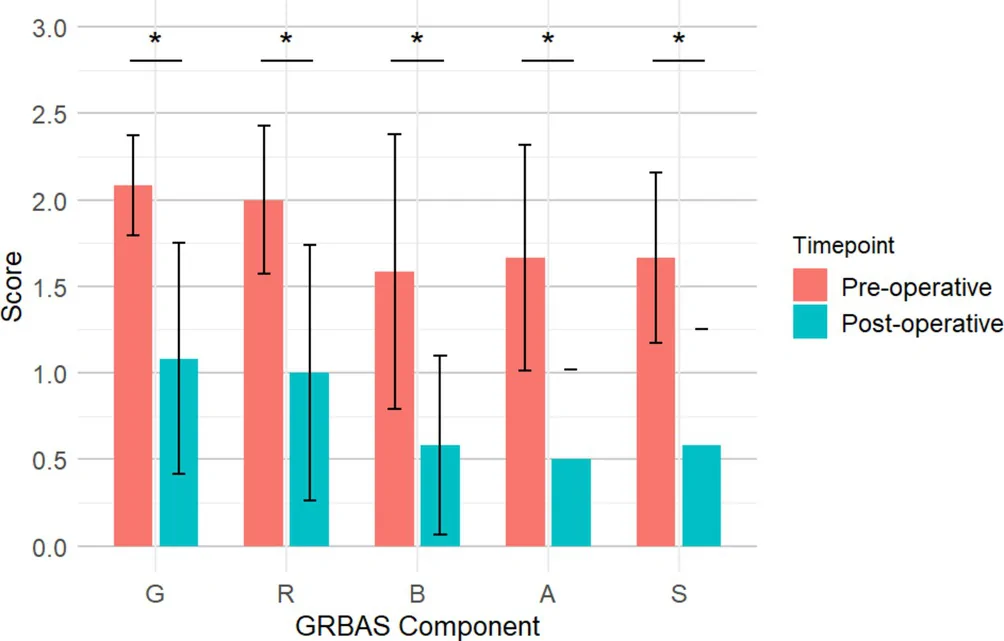
Comparison of composite pre‐ and postoperative GRBAS scores for all patients (*n* = 12) reported as a mean of patient scores ±1 SD; Wilcoxon signed rank test for comparison (*p ≤ 0.05).

Patients' subjective vocal performance was assessed using the VHI‐10. The mean preoperative score was 25.75 ± 9.36. Postoperatively it decreased significantly to 14.92 ± 9.55 (Wilcoxon signed‐rank, *p* = 0.0093) as seen in Figure 3.

**FIGURE 3:**
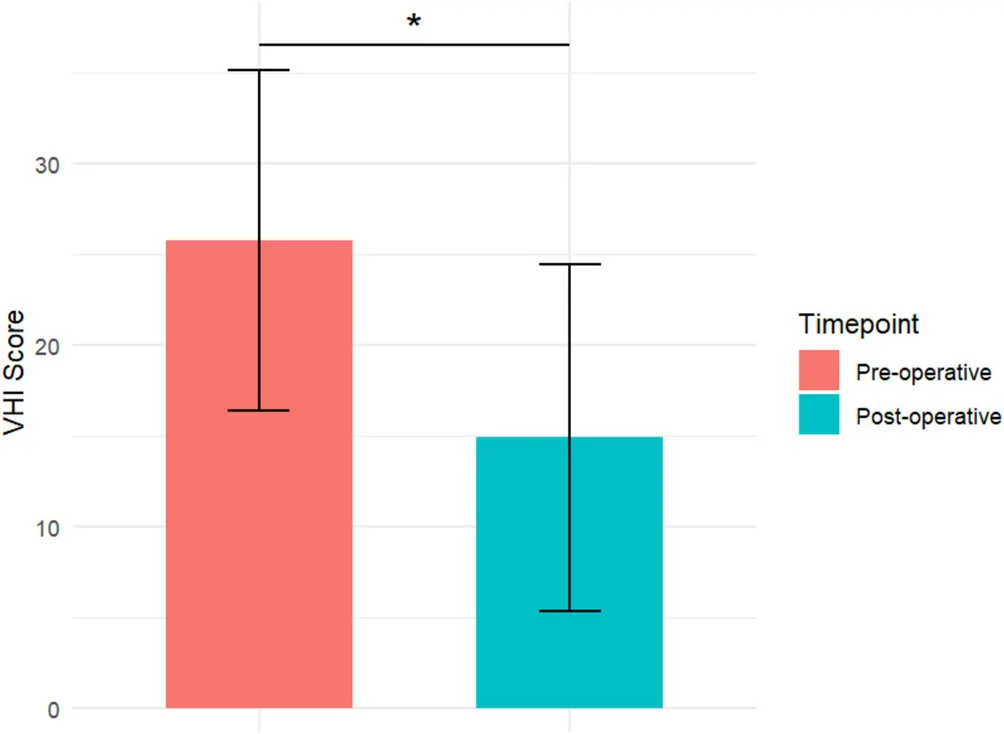
Pre‐ and postoperative VHI‐10 score from patients (*n* = 12); Wilcoxon signed rank test for comparison (*p ≤ 0.05).

### 3.3 Postoperative Implant Adjustments and Complications

Two patients (16.7%) required postoperative adjustment of their implant via saline injection to correct under‐medialisation. One adjustment, an addition of 0.25 mL, was performed in clinic 8 months postoperatively, while the other, an addition of 0.3 mL, was performed in theatre under local anaesthetic due to a non‐palpable injection port at 3 months. Voice quality was assessed subjectively during both procedures. Both demonstrated an improvement in GRBAS score and reduction in VHI on the final follow‐up compared to initial scores, and optimal medialisation on FNE assessment.

No intraoperative or postoperative complications using Clavien Dindo classification were noted and no patients were readmitted to hospital [17]. All patients received prophylactic co‐amoxiclav for 1 week postoperatively.

## 4 Discussion

The current evaluation of the APrevent VOIS Vocal Implant System demonstrated a significant improvement in all components within the GRBAS scale as well as patient‐reported outcomes. All patients reported a noticeable improvement in voice quality and quality of life, exceeding the minimal clinically important difference (a ≥ 5‐point reduction in VHI‐10) defined for vocal fold paralysis by Young et al. (2018) [18]. These benefits were evident at outpatient follow‐up appointments and aligned with the findings of Burk et al. (2025), who also reported significant improvements in GRBAS and VHI‐10 scores in their 12‐patient series [19].

In the wider literature on vocal cord medialisation, a variety of outcome measures have been employed, complicating direct comparison between the outcomes of medialisation with VOIS compared to IL or other thyroplasty implant types. Nonetheless, among studies reporting GRBAS and VHI‐10 scores, the VOIS implant performed comparably to silicone, titanium, and Gore‐Tex designs [20, 21, 22].

Across implant types, several studies report significant postoperative improvements in the majority of GRBAS parameters. One study exploring the use of a silicone device demonstrated sustained improvement in GRBAS values maintained at 5 years after implantation [20]. Similarly, statistically significant differences in composite GRBAS scores were achieved after Gore‐Tex medialisation thyroplasty [21]. In a direct comparison of silicone and titanium implants, Van Ardenne et al. found a comparable significant improvement in most components of the GRBAS scale for both materials [22].

However, the ‘roughness’ component of the GRBAS scale shows more variable improvement across the literature, which appears to reflect patient factors rather than implant choice. For one, in Van Ardenne et al.'s analysis, ‘roughness’ was not significantly improved after surgery and could not reliably differentiated outcomes between the two implant materials [22]. Burk et al. reported similar findings specifically for the VOIS implant, whereby persistent vocal roughness was observed immediately after medialization thyroplasty with VOIS in patients with long‐standing vocal cord paralysis. They suggested that this may reflect chronic compensatory laryngeal changes that require additional speech and language therapy rather than limitations of the implant itself [19]. This finding is therefore not unique to the VOIS implant but is consistent with the broader literature showing that roughness can be the most resistant GRBAS parameter to normalise after medialisation, regardless of implant type.

Overall, voice assessment relies on multiple complementary scales. Evaluating surgical success using a single parameter provides limited insight; rather, consistent improvements across both perceptual and patient‐reported outcomes support VOIS as an effective and promising alternative to conventional medialisation techniques.

Preoperative imaging has been employed in some studies to guide implant design and surgical planning. For instance, Frizzarini et al. (2012) used cadaveric laryngeal scans to determine optimal implant dimensions, while Ho et al. (2023) incorporated CT and MRI data into the development of the VOIS prototype [12, 23]. Imaging has also been used postoperatively to confirm implant position in other medialisation systems [24, 25]. In this series, imaging was solely used for diagnostic purposes rather than for preoperative planning.

This study has some limitations, including a small sample size and relatively short follow‐up period. Although long‐term regular follow‐up would showcase the sustained benefits of VOIS in treating UVCP, this was not feasible within the local care pathway, where routine postoperative assessment is not conducted unless patients are referred with complications or persistent unsatisfactory voice outcomes. Hence, larger, multi‐centre studies with extended prospective assessment are required to assess long‐term outcomes and reproducibility. Two patients required postoperative adjustment of implant volume to optimise the voice quality. In one case, the access port was easily palpated in the clinic; in the other, revision under local anaesthetic was performed in the operating room due to difficulty locating the port. Although postoperative adjustability is a key advantage of VOIS over conventional thyroplasty, further experience will determine how reliably these adjustments can be performed in outpatient settings. The two patients in question were only subjectively assessed prior to the adjustment of their implants; following this, they were not referred back to the department with complications or for further saline volume corrections. Objective acoustic and aerodynamic parameters such as jitter, shimmer, and maximum phonation time were not recorded in this study, which limits the assessment of quantitative voice outcomes. Also, blinding of voice assessors was not feasible given the clinical nature of the intervention.

## 5 Conclusion

This series demonstrates that medialisation thyroplasty using the APrevent VOIS system provides significant objective and subjective improvement in vocal function following unilateral vocal cord paralysis. Further work with larger cohorts and extended follow‐up is required to evaluate the durability of these benefits, the need for secondary adjustments, and long‐term device performance. Ongoing clinical use will expand the evidence base and clarify the implant's role within contemporary management of UVCP.

## Author Contributions

All authors S.K., M.K., C.G., Y.D., G.‐M.H., R.B. made substantial contributions to the conception or design of the work or the acquisition, analysis, or interpretation of data for the work, drafted the work or revised it critically for important intellectual content, gave final approval of the version to be published, and agree to be accountable for all aspects of the work in ensuring that questions related to the accuracy or integrity of any part of the work are appropriately investigated and resolved.

## Funding

The authors have nothing to report.

## Conflicts of Interest

One of the authors (Guan‐Min Ho) is the founder and/or holds an affiliation with APrevent Biotech GmbH, the manufacturer of the VOIS implant. This potential conflicts of interest has been fully disclosed. The author had no role in the outcome assessment or data analysis. All other authors declare no conflicts of interest or financial relationships related to this study.

## Data Availability

The data that support the findings of this study are available on request from the corresponding author. The data are not publicly available due to privacy or ethical restrictions.
